# TMJ metastasis: A unusual case report

**DOI:** 10.1186/1746-160X-4-8

**Published:** 2008-06-04

**Authors:** Roberto Boniello, Giulio Gasparini, Giuseppe D'Amato, Alessandro Di Petrillo, Sandro Pelo

**Affiliations:** 1 Department of Maxillo Facial Surgery, Catholic University Medical School, Rome, Italy

## Abstract

The metastases to the TMJ from a primary lung tumour is a very rare occurrence.

This case is unusual in several aspects, as the non-reducible dislocation of the TMJ was the first clinical manifestation of the tumour. CT staging showed that this secondary tumour in the condyle was the only bone metastasis.

## Introduction

Metastases involving the TMJ are rare, and only 40 cases have been reported in the international literature [1]. The primary site of the carcinoma was the breast in 9 cases, the lung in 9, the prostate in 5 and the rectum in 3 [2], followed by the liver, cardia [3], uterus and pancreas (1 case each). In 2 cases the primary tumour was a melanoma [4], the nose being the primary site in one case and the hallux in the other. In 6 cases, the primary site was not identified. As a rule, TMJ metastases manifest clinically even years after the onset of the primary tumour. In this study, we present a very unusual case, unique in the literature, in which the non-reducible dislocation of the mandible was the first clinical manifestation of pulmonary adenocarcinoma.

## Case presentation

**Figure 1:**
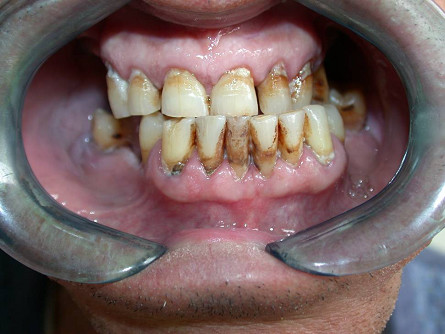
The oral examination revealed Class III malocclusion and left mandibular deviation.

The patient, a 60-year-old male, underwent extraction of the lower first right molar. From that moment he suffered from right TMJ pain. At first his symptoms were associated to the intervention. Following this episode, the patient reported limitation of mandibular movements, with reduced mouth opening, inability to perform lateral movements, right crossbite and edge-to-edge occlusion. The oral examination revealed Class III malocclusion and left mandibular deviation. (Fig. 1) On palpation the glenoid cavity was found to be empty.

An orthopanoramic X-ray showed dislocation of the right condyle.

After unsuccessful attempts to reduce the dislocation on an outpatient basis, it was decided to perform manual reduction under general anaesthesia, without positive result. Consequently an open reduction under general anaesthesia was performed.

During routine preoperative exams, the chest X-ray showed an opaque area with microcalcifications. Given the high level of significance of the temporomandibular symptoms and the fact that the patient's cardiorespiratory parameters and blood tests were normal, after consulting with pulmonologists we decided to perform surgery on the condylar area and to postpone diagnosis and treatment decisions involving the lung until immediately after surgery

**Figure 2:**
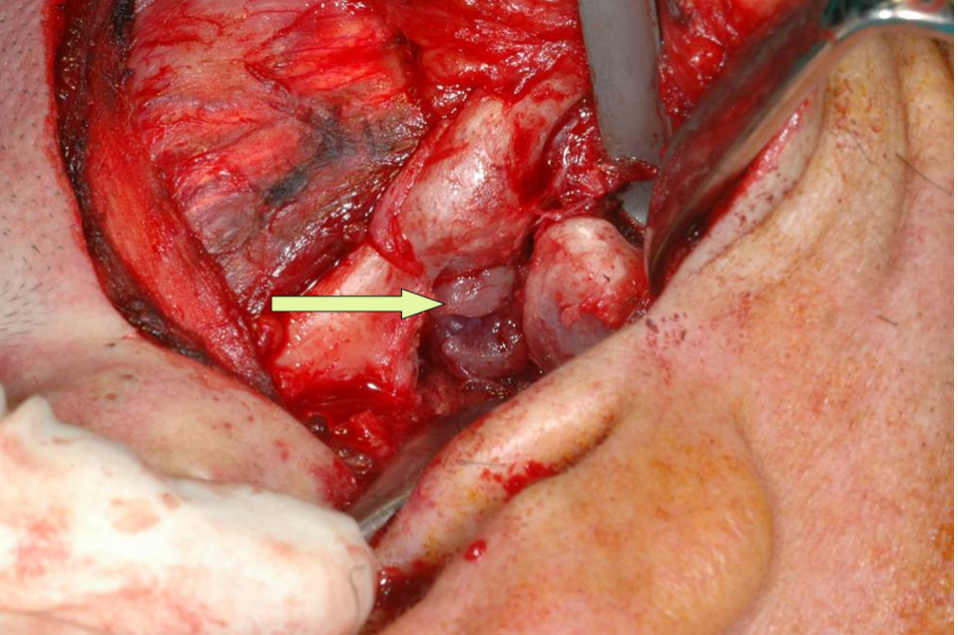
**Intraoperative view.** The condyle was displaced past the eminence. The mass occupied the intra-articular space.

The joint capsule was accessed through a pretragal incision, observing that the condyle was displaced past the eminence. An unsuccessful attempt was made to perform bimanual reduction. It was finally observed that there was a mass that completely occupied the intra-articular space. The tumour was thus circumscribed and resected, after which the dislocation was successfully reduced manually; Deutrey's procedure was then performed to constrict the condyle. (Fig. 2)

**Figure 3:**
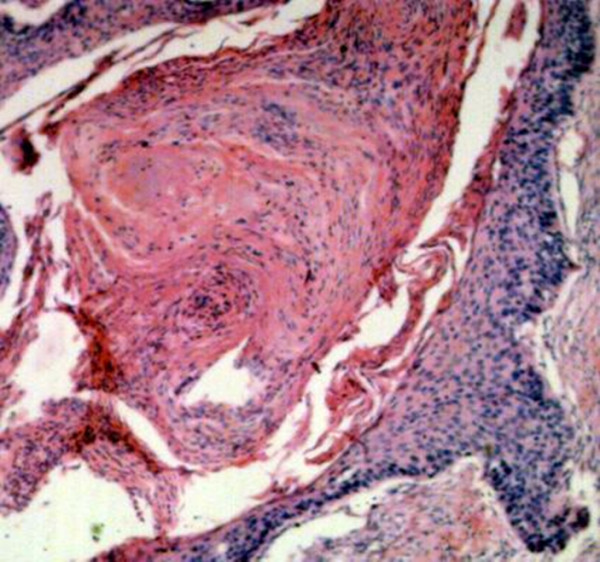
The histological examination showed that the lesion was malignant and had metastasised from a primary lung tumour.

The histological examination showed that the lesion was malignant and had metastasised from a primary lung tumour. (Fig. 3)

A post-operatory chest X-ray appeared within the normal range, and only a post-operatory spiral CT scan succeeded in identifying the primary tumour The patient was referred to the pulmonary and oncology departments. Based on bronchoscopy and biopsy results, pulmonary adenocarcinoma was diagnosed. Given the presence of distant metastases, the patient was classified as Stage IV (TNM) [5]. However, a total-body CT scan revealed that the condylar lesion was the only distant metastasis. The patient underwent a cycle of radiotherapy, but died 6 months later.

## Discussion

Tumours of the TMJ – both benign and malignant – are rare but difficult to diagnose [6]. In view of the fact that, at onset, they resemble common temporomandibular disorders, they can often mislead the specialist in formulating correct diagnostic suspicions. Therefore, in patients who do not respond to treatment it is appropriate to reconsider the diagnosis and include the suspicion of cancer in the differential diagnosis.

This case is particularly interesting because it not only involves an uncommon secondary site, but onset was extremely atypical, as the clinical manifestation of bone metastasis preceded that of the primary carcinoma. What is even more significant is that the nature of the tumour involved was a non-small-cell carcinoma.

The mandible and thus the condyle are unusual sites for metastases. Metastases to the entire maxillomandibular complex represent just 1% of metastatic cancers and 1% of all tumours in this area [7]. Metastases seem to prefer the marrow of haematopoietically active bone tissue, as this red marrow is richer in sinusoids that allow neoplastic clones to colonize and proliferate [8]. However, the mandible is not a haematopoietic site, particularly in older patients.

## Conclusion

Therefore, the case described here is a very significant example of the diagnostic challenges that TMJ lesions can represent. The authors underline the need to include condylar tumours in the differential diagnosis when the symptoms do not respond to treatment. A CT or MRI scan of the TMJ should be part of the diagnostic procedure.

The case presented is the first example in literature of the clinical onset of lung cancer with a non-reducible dislocation of the temporomandibular joint.

## Competing interests

The authors declare that they have no competing interests.

## Consent

Written informed consent was obtained from the patient for publication of this case report and any accompanying images. A copy of the written consent is available for review by the Editor-in-Chief of this journal.

## Authors' contributions

All the authors were involved in examination of the patient as well as in writing and reviewing the manuscript.
